# Practical guidance for echocardiography for cancer therapy-related cardiac dysfunction: 2026 focused update

**DOI:** 10.1007/s12574-026-00731-4

**Published:** 2026-05-03

**Authors:** Hidekazu Tanaka, Yuko Fukuda, Hirotsugu Yamada, Sakiko Miyazaki, Jiro Sakamoto, Masao Daimon

**Affiliations:** 1 Division of Cardiology, Department of Internal Medicine, Kindai University Faculty of Medicine, Osaka, Japan; 2 Department of Onco-Cardiology/Cardiovascular Medicine, The Cancer Institute Hospital of Japanese Foundation for Cancer Research, Tokyo, Japan; 3 Department of Community Medicine for Cardiology, Tokushima University Graduate School of Biomedical Sciences, Tokushima, Japan; 4 Department of Cardiovascular Biology and Medicine, Juntendo University Graduate School of Medicine, Tokyo, Japan; 5 Department of Cardiology, Tenri Hospital, Tenri, Japan; 6 Department of Cardiology, Tokyo Women’s Medical University, Tokyo, Japan

**Keywords:** Cancer therapy–related cardiac dysfunction, Echocardiography, Global longitudinal strain, Cardio-oncology

## Abstract

**Background:**

Cancer therapy–related cardiac dysfunction (CTRCD) has become an important clinical issue with advances in cancer treatment and improved patient survival. The Japanese Society of Echocardiography previously published practice guidance in 2020. The present document provides an updated revision reflecting recent developments in cardio-oncology.

**Methods:**

This guidance was developed based on contemporary evidence, including the 2022 European Society of Cardiology cardio-oncology guidelines, recent clinical studies, and advances in echocardiographic and multimodality imaging technologies.

**Results:**

Left ventricular ejection fraction (LVEF) and global longitudinal strain (GLS) are emphasized as essential parameters for diagnosing and monitoring CTRCD. The document provides standardized protocols for echocardiographic evaluation before, during, and after cancer drug therapy, as well as recommendations for long-term surveillance following radiotherapy. It also addresses cardiovascular complications associated with immune checkpoint inhibitors, particularly myocarditis, and highlights the importance of measurement accuracy, quality control, artificial intelligence, and three-dimensional echocardiography in clinical practice.

**Conclusions:**

This updated guidance offers practical and evidence-based recommendations for echocardiographic assessment in cardio-oncology, aiming to facilitate early detection of cardiotoxicity and optimize multidisciplinary management.

## Myocardial dysfunction associated with cancer therapy: overview

### Background of the revision

In 2019, the Japanese Society of Echocardiography created the "Practical guidance for echocardiography for cancer therapeutics‑related cardiac dysfunction" (2nd edition, 2020 [1], hereinafter referred to as the “Guidance”). Thereafter, cardio-oncology guidelines were published by the European Society of Cardiology (ESC) in collaboration with the European Hematology Association (EHA), the European Society for Radiotherapy and Oncology (ESTRO), and the International Cardio-Oncology Society (IC-OS) in 2022 [2] and jointly by the Japanese Society of Cardiovascular Oncology and the Japanese Society of Medical Oncology [3] in 2023. Furthermore, new regimens of cancer drug therapies, including immune checkpoint inhibitors (ICIs), were launched, leading to rapidly increasing cases where clinicians encounter new pathological conditions such as myocarditis. In this situation, echocardiography remains a critical tool that plays a gatekeeper role in detecting myocardial disorders, thus motivating us to revise the Guidance. Note that the title of the Guide was changed to the " Practical guidance for echocardiography for cancer therapy-related cardiac dysfunction" in accordance with the term cancer therapy-related cardiac dysfunction (CTRCD) in Japan’s cardio-oncology Guidelines [3].

### Definitions of terms in cardiac dysfunctions related to cancer therapy

In the field of echocardiography, CTRCD has long been used, reflecting historical milestones, including the establishment of evidence supporting the early diagnosis of CTRCD using global longitudinal strain (GLS) in adults with drug-induced myocardial disorders by anthracycline drugs and anti-HER2 antibody drugs (patients receiving drug therapy for breast cancer, malignant lymphoma, certain leukemias, sarcomas, etc.). In clinical settings, on the other hand, the same term may be used to include cardiovascular disorders associated with non-drug treatments. To avoid confusion in terminology, however, the Guide handles radiotherapy-induced cardiovascular disorders as radiation-induced heart disease (RIHD) and myocarditis associated with immune checkpoint inhibitors (ICIs) as ICI-related myocarditis, a manifestation of immune-related adverse events (irAEs). Differing from CTRCD pathologically and involving unique managerial and therapeutic policies, these conditions should be handled distinctly. In reality, however, we sometimes encounter cases where it is difficult to accurately identify the causative drugs for CTRCD. Based on the ESC Cardio-Oncology Guidelines and other sources, Table 1 shows cancer treatments that, in addition to CTRCD and ICI-induced myocarditis, can be complicated by vascular diseases (venous thrombosis, arterial thrombosis, etc.), hypertension, and arrhythmias.

**Table 1:** Cancer treatments that induce cardiovascular toxicity

Cancer treatments that can cause cardiovascular disease	Major pathological conditions (Although some drugs are associated with specific adverse events, not all agents within the same class necessarily cause the adverse events listed below. Since the presence/absence and frequency of cardiovascular toxicity vary among different drugs, guidelines and drug information should be checked.)
Anthracycline drugs	These are representative causative drugs for CTRCD
Anti-HER2 therapy	A lifetime dosage is specified; it is important to check for history of prior treatment. This is used mainly for breast cancer, malignant lymphoma, and sarcoma
Fluoropyrimidine drugs	These are representative causative drugs for CTRCD
VEGF inhibitors (angiogenesis inhibitors)	In many patients, cardiac function is restored after drug cessation, but recovery usually takes 2–3 months
Second- and third-generation BCR/ABL tyrosine kinase inhibitors	Some adverse events are known to be associated with coronary vasospastic angina
BTK inhibitors (Bruton’s tyrosine kinase inhibitors)	These can cause hypertension, arterial thrombosis, venous thrombosis, myocardial infarction, QT prolongation, and heart failure (myocardial dysfunction)
Proteasome inhibitors	These include VEGF inhibitors, VEGF receptor inhibitors, and multi-kinase inhibitors; they are associated with different frequencies of adverse events
BRAF/MEK inhibitors	These are known to be associated with adverse events such as pulmonary hypertension, pericardial effusion, pleural effusion, hypertension, atrial fibrillation, QT prolongation, vascular toxicity, heart failure, hyperglycemia, and dyslipidemia
Immune checkpoint inhibitors (ICIs)	In addition to pulmonary hypertension, dasatinib can cause pericardial effusion and pleural effusion
Androgen deprivation (blockade) therapy for prostate cancer	First-generation ibrutinib is known to be associated with atrial fibrillation, hypertension, heart failure, and QT prolongation
Endocrine therapy for breast cancer	Second-generation acalabrutinib can be associated with atrial fibrillation
CDK4/6 inhibitors	These can be associated with adverse events such as hypertension, heart failure, atrial fibrillation, hyperglycemia, dyslipidemia, venous thrombosis, and pulmonary hypertension
ALK inhibitors	Reports on the utility of GLS are available
EGFR inhibitors	In the treatment of multiple myeloma, various drugs are used in addition to proteasome inhibitors, such as immunomodulatory drugs, antibody drugs, and cytotoxic anticancer drugs, which have different cardiovascular toxicities
CAR-T therapy	In addition, multiple myeloma can be complicated by AL amyloidosis
Radiotherapy	This can cause hypertension, heart failure, hyperglycemia, dyslipidemia, bleeding, venous thrombosis / pulmonary thromboembolism, etc
Hematopoietic stem cell transplantation	In principle, RAF inhibitors and MEK inhibitors are used in combination

### Diagnostic criteria and severity classification of CTRCD

The ESC Guidelines proposed a new severity classification for CTRCD (Table 2) [2]. Because the severity ratings directly inform decision-making for the continuation or switching of cancer treatment and for CTRCD treatment (such as whether cardioprotective medications can be discontinued in the future), the Guidelines emphasize the importance of clarifying the severity of the disease at the same time as diagnosing CTRCD. The severity of asymptomatic CTRCD, in particular, is determined mainly by echocardiography because its ratings are based on left ventricular ejection fraction (LVEF) and GLS. Note that the Common Terminology Criteria for Adverse Events (CTCAE) are used for evaluating adverse events in oncology [4]. An “adverse event” as evaluated in the CTCAE refers to any unwanted sign experienced by the patient and differs from an “adverse reaction” as it is not necessarily related to the treatment or procedure in terms of causality. Severity is determined in Grades 1 to 5 as follows: Grade 1 represents a mild adverse event (asymptomatic or mild symptom, minor laboratory finding) that does not require therapeutic intervention, often allowing cancer treatment to be continued. Grade 2 represents a moderate adverse event (symptomatic but only limited to age-appropriate limitations in daily living activities other than self-care) requiring minimal/localized/non-invasive treatment. Cancer treatment may be postponed or interrupted. Grade 3 represents an adverse event that is severe or medically significant but not immediately life-threatening, requiring inpatient hospitalization or prolongation of existing hospitalization. Ongoing cancer treatment is interrupted or terminated (including regimen changes). Grade 4 is a life-threatening adverse event requiring emergency treatment. Cancer treatment is terminated. Grade 5 is death due to an adverse event. Note that CTCAE Ver 6.0 updated the section on cardiac disorders, left ventricular dysfunction, to establish the following criteria: Grade 1: A left ventricular ejection fraction (LVEF) ≥ 50% and meeting at least one of the following requirements: 1) A new decrease in GLS exceeding 15% from baseline and 2) a new increase in a cardiac biomarker. Grade 2: A LVEF of 40–49% and meeting at least one of the following requirements: 1) A decrease in LVEF by ≥ 10%, 2) a new decrease in GLS exceeding 15% from baseline, and 3) a new increase in a cardiac biomarker. Grade 3: LVEF has newly decreased to below 40%. Grades 4 and 5 are not defined.

**Table 2:** Severity classification of CTRCD

Symptomatic	Very severe	Heart failure requiring inotropic or circulatory support, or requiring consideration for heart transplantation
	Severe	Cases requiring hospitalization for heart failure
	Moderate	Cases requiring intensification of diuretics and heart failure medications at an outpatient clinic
	Mild	Mild symptoms of heart failure are present but not requiring treatment intensification
Asymptomatic	Severe	Cases of new manifestation of LVEF < 40%
	Moderate	Either [1] or [2] [1] LVEF decreased newly by ≥ 10% to 40–49% [2] LVEF decreased by < 10% to 40–49% and any of the following cases: - A new relative reduction of GLS from baseline (ΔGLS) exceeding 15% - A new elevation of cardiac biomarkers (*)
	Mild	LVEF ≥ 50% and ΔGLS > 15% and/or a new elevation of cardiac biomarkers (*)

Adapted from Reference 2. (*) Elevation of cardiac biomarkers: elevation of cTnI or cTnT above the 99th percentile, BNP ≥ 35 pg/mL, NT-proBNP ≥ 125 pg/mL, or a new significant elevation from baseline (beyond the biological or analytical error of the assay used)

### Frequency and prognosis of CTRCD and significance of early diagnosis

When using a regimen involving concurrent administration of anthracycline and trastuzumab (1995–1997), left ventricular dysfunction and heart failure occurred at extremely high rates of 27% and 16%, respectively [5]; it is recommended that this regimen be avoided. A meta-analysis of randomized controlled trials (RCTs) conducted between 1997 and 2019 to evaluate the effects of cardioprotective drugs on anthracycline drugs showed that LVEF decreased by a mean of 5.4% (3.5–7.3%) from baseline to 6 months; the doxorubicin-equivalent dose of the anthracycline drugs was 385 mg/m^2^.[6] This meta-analysis includes studies on patients with hematological cancer in addition to those with breast cancer. Anthracycline drugs began to be used in the 1960s, and many cases of cardiotoxicity were reported in the 1970s. Accordingly, recent regimens take cardiotoxicity into consideration, and this appears to reflect the above findings. CTRCD due to anthracycline drugs is said to develop in 90% of its recipients within one year after treatment completion [7]. Furthermore, research has shown that early intervention in heart failure treatment in CTRCD cases increases the likelihood of recovery of cardiac function [8]. Therefore, early diagnosis and treatment intervention for CTRCD are important.

### Risk stratification and management of CTRCD

The ESC Guidelines propose a risk factor assessment method for CTRCD [2]. Exposure to cardiotoxic substances equals a risk of heart failure (heart failure stage A) [9]; a recommended frequency of echocardiographic follow-up is specified for each regimen. As emphasized in the ESC Guidelines, it is too late to address CTRCD after its onset; importance should be placed on baseline assessment at (or before) the start of cancer treatment, management (surveillance) during cancer treatment, and survivorship care after the completion of cancer treatment. Although management is not necessarily required in all cases, it is necessary to consider a reasonable multidisciplinary approach to intervening patients at high risk of undergoing cardiotoxic cancer treatments in line with the specific circumstances of each facility.

## Diagnosis of CTRCD

### Echocardiography

Echocardiography is a non-invasive procedure that involves no radiation exposure, can be performed repeatedly, and is widely used in general clinical practice. This method enables measurement of LVEF, which is used in the diagnostic criteria for CTRCD. Therefore, it is the most frequently used imaging technique for evaluating cardiac function before cancer treatment with potentially cardiotoxic drugs or radiotherapy and for monitoring cardiac function during and after cancer treatment [10, 11]. In addition, this method is not only used to evaluate the sizes of the left and right ventricles and cardiac functions (systolic and diastolic), but is also frequently used to diagnose organic cardiovascular diseases such as ischemic heart disease, valvular heart disease, large vessel disease, pulmonary hypertension (PH), and pericardial disease, as well as cardiac tumors (primary and metastatic), and to assess their severity [12]. Hence, echocardiography is widely useful not only for diagnosing CTRCD but also for diagnosing cardiac disease in cardio-oncology practice.

#### Left ventricular contractility

##### LVEF

This is included in the definition of cardiac dysfunction in CTRCD, requiring quantitative assessment that is accurate and reproducible.

##### Method of measurement

The disk method is recommended, in which the left ventricular endocardial border is traced at end-systole and end-diastole in the apical four- and two-chamber views to calculate LVEF. However, the disk method poses some problems in measuring LVEF: its reproducibility is not always high. Strategies to address these issues in clinical practice are discussed in the section entitled ‘Echocardiographic Protocol for Patients Undergoing Cancer Drug Therapy. The American Society of Echocardiography (ASE) and the European Association of Cardiovascular Imaging (EACVI) jointly recommend measuring the LVEF using three-dimensional (3D) echocardiography [13]. Advances in ultrasound diagnostic equipment and automated measurement technology have made it possible to obtain more accurate and more reproducible measurements of left ventricular volume than those with the disc method, using 3D echocardiography, provided that good image data are acquired [14]. However, only a few facilities routinely perform 3D echocardiography, and image quality issues limit the number of applicable cases [15], and the normal values of LVEF using 3D echocardiography, unlike those with the disc method [16], have no defined cutoff values for CTRCD. For these and other reasons, it is not recommended for routine clinical practice in CTRCD at present. While typical CTRCD presents with diffuse left ventricular wall motion abnormalities, cancer drug therapy increases the risk of causing ischemic heart disease [17], hence sometimes leading to left ventricular local wall motion abnormalities. Because left ventricular wall motion does not necessarily decrease uniformly and accurate quantitative assessment of LVEF is required, and for other reasons, visual assessment (“eyeball” estimation of EF) and the Teichholz method by M-mode imaging are considered insufficient.

##### Reference value

In the ASE Guidelines for Chamber Quantification published in 2005, the lower limit of normal LVEF values was 55% [18]. Therefore, the LVEF was set at 55% for the diagnostic criteria for CTRCD. Taking newer study results into account, the latest version of the guidelines published in 2015 set the lower limit of normal LVEF values at 53% [16]. In the ESC position paper, CTRCD is defined as "a condition in which LVEF has decreased to less than the lower limit of normal by more than 10 percentage points from baseline," [19, 20] hence as a condition where "LVEF that has decreased to less than 53% by more than 10 percentage points from baseline." However, taking into account the likely measurement errors in LVEF and the variability of normal values of LVEF, this Guidance defines CTRCD as "a condition in which LVEF has decreased to < 50% by more than 10 percentage points from baseline." Hence, “LVEF 57% → 46%” meets the criteria, whereas “LVEF 57% → 49%” and “LVEF 65% → 54%” do not. The Cardio-Oncology Practice Handbook edited by the Japanese Onco-Cardiology Society [21] and the Onco-cardiology Guidelines by the Japanese Society of Medical Oncology [3] specify the lower limit of LVEF to be 50%; currently, CTRCD is often diagnosed when the LVEF value falls below 50% in connection with cancer treatment. Because this reference value is likely to change due to compiled evidence from advanced research, its updates are necessary. In addition, because the condition is not clearly identifiable with a single numerical figure, it is important to take flexible measures in clinical practice, such as shortening the duration of follow-up, when LVEF shows a declining trend.

##### GLS

The measurement error for LVEF with the disk method is approximately 10% [13, 22], which is comparable to the 10% reduction from baseline used in the diagnostic criteria for CTRCD, hence posing a major problem. Due to reproducibility issues, slight changes in LVEF values do not necessarily represent true changes; in recent years, GLS measured using the speckle-tracking method has been increasingly used [23]. As a highly reproducible indicator capable of detecting myocardial disorders more sensitively than LVEF, GLS is recommended not only in cardiovascular guidelines in Europe and the United States [13, 19] but also in the guidelines of the American Society of Clinical Oncology. Accordingly, the Onco-cardiology Guidelines by the Japanese Society of Medical Oncology [3] weakly recommend that GLS be measured during periodic echocardiography in cancer drug therapy. Note that in the present Guidance, GLS is displayed with absolute values. For facilities where GLS cannot be measured using the speckle tracking method, evaluations are recommended in terms of mitral annular plane systolic excursion (MAPSE) calculated using the M-mode, an index of left ventricular longitudinal myocardial contractility as with GLS, or systolic mitral annular motion velocity (S’) calculated using the tissue Doppler imaging [13]. However, for both MAPSE and S’, no cutoff value is available to detect cardiotoxicity due to cancer drug therapy, like for GLS; therefore, the interpretation remains limited to latent left ventricular myocardial disorder being suspected only with a marked reduction compared with the previous and baseline values.

##### Method of measurement

GLS is analyzed from video data from three apical views (longitudinal, two-chamber, and four-chamber cross-sectional views) over the cardiac cycle using the two-dimensional speckle tracking method, either with software built into the device or a dedicated analysis computer. In common software, the GLS is obtained by averaging the peak values of the global strain curve created from the standard three cross-sectional views of apical approaches. Although GLS is sometimes output as a minus value, all values are expressed as absolute (positive) values (plus values) in the present Guidance. Because strain measurement involves variation in ROI settings (endocardial side, full thickness, etc.) and tracking methods among different systems (vendors), it is strongly recommended that the clinical course be followed using the same device and software.

##### Reference value

The normal GLS values for Japanese people are reported by Takigiku et al. [24] Table 3 summarizes studies evaluating event prediction using GLS measured before the initiation of cancer therapy [25–33]. These studies employed GLS cutoff values of 15–19%. Since 18% is often used as the lower limit of normal GLS, values below 16% are considered to indicate a high risk, and 16–18% are considered to represent a borderline. In cases where GLS has decreased by ≥ 15% from baseline after administration of an anticancer drug (e.g., 25% → 21%, a relative decrease of 16% meeting the criteria; 25% → 22%, a relative decrease of 12% not meeting the criteria), it should be determined that cardiotoxicity (any latent left ventricular myocardial disorder) due to cancer drug therapy has begun, even without any observed significant decrease in LVEF [13, 19]. On the other hand, if the relative decrease in GLS is less than 8% after cancer drug therapy, it should be determined that there is no cardiotoxicity due to cancer drug therapy (no latent left ventricular myocardial disorder) [13].

**Table 3:** Recommended timing of echocardiography during cancer drug therapy

	Nonproprietary name (abbreviation)	Trade name	Recommended time points for echocardiography
Anthracyclines	Doxorubicin	Adriamycin	Low risk: before treatment, 4th cycle, 1 year later Moderate risk: before treatment, 4th cycle, 1 year later High risk: before treatment, 2nd, 4th, and 6th cycles, 3 months later, 1 year later Recommended for low- and moderate-risk patients when the cumulative doxorubicin dose exceeds 250 mg/m^2^
	Pirarubicin	Therarubicin	
	Farmorubicin	Epirubicin, Farmorubicin	
	Doxorubicin hydrochloride ribosomal preparation	Doxil	
Anti-HER2 drugs	Trastuzumab	Herceptin	Low or moderate risk: before treatment, every 3 months during treatment High risk: before treatment, every 3 months during treatment
	Trastuzumab-emtansine	Kadcyla	
	Trastuzumab-deruxtecan	Enhertu	
	Pertuzumab	Perjeta	
	Pertuzumab-trastuzumab combination	Phesgo	
Angiogenesis inhibitors	Bevacizumab	Avastin	Low risk: before treatment Moderate risk: before treatment, every 4 months during treatment High risk: before treatment, every 3 months during treatment In cases at very-high risk, an additional examination at 3 weeks is recommended
	Aflibercept	Zaltrap	
	Ramucirumab	Cyramza	
	Sunitinib	Sutent	
	Sorafenib	Nexavar	
	Pazopanib	Votrient	
	Axitinib	Inlyta	
	Regorafenib	Stivarga	
	Vandetanib	Caprelsa	
	Ponatinib	Iclusig	
	Cabozantinib	Cabometyx	
	Lenvatinib	Lenvima	
Immune checkpoint inhibitors	Ipilimumab	Yervoy	Before initiation of treatment
	Nivolumab	Opdivo	
	Pembrolizumab	Keytruda	
	Avelumab	Bavencio	
	Atezolizumab	Tecentriq	
	Durvalumab	Imfinzi	
	Tremelimumab	IMJUDO	
	Cemiplimab	LIBTAYO	

#### Left ventricular diastolic function

Data demonstrating the usefulness of left ventricular diastolic function indices are limited for diagnosing CTRCD, following up the patient, and predicting the prognosis. However, systematic assessment of left ventricular diastolic function and subsequent assessment of left ventricular filling pressure should be performed as part of ordinary routine examination in accordance with the existing guidelines [34]. Because the load conditions fluctuate due to fluid replacement and adverse reactions (vomiting, diarrhea, etc.) associated with cancer drug therapy, caution is required when using E/e’ to estimate left ventricular filling pressure. Elevated left ventricular filling pressure suggests heart failure; therefore, it is desirable, if such a finding is observed, to consult a cardiologist even in the absence of apparent symptoms.

#### Right ventricular function and pulmonary arterial pressure

Only limited data are available regarding the usefulness of right ventricular function assessment in clinical practice for CTRCD. When using therapeutics that carry a risk of pulmonary arterial hypertension, such as tyrosine kinase inhibitors, including dasatinib, or when cancer-related thrombosis is suspected, evaluation of right ventricular systolic function is important. Echocardiography is also useful for diagnosing a cancer-related disease characterized by acute PH, known as pulmonary tumor thrombotic microangiopathy (PTTM). If echocardiography reveals right ventricular enlargement or decreased right ventricular systolic function or suggests PH, consultation with a cardiologist is recommended.

### Other means of imaging

#### Cardiac nuclear medicine (myocardial scintigraphy)

LVEF can be measured using multi-gate electrocardiogram-gated cardiac pool imaging, a method in which ^99 m^ technetium is adsorbed to red blood cells, which are imaged using a gamma camera in synchronization with electrocardiograms, and the radioactivity within the cardiac chambers is measured. This method can be used to monitor cardiac function during chemotherapy [35] and is indicated for Class 1A in the American Heart Association (AHA) / American College of Cardiology (ACC) Guidelines [36]. A report concludes this is useful for the early detection of cardiotoxicity [37]. While it offers high reproducibility and is useful when LVEF cannot be evaluated by echocardiography, it is disadvantageous due to radiation exposure and high medical costs.

#### Cardiac MRI

Cardiac MRI is currently considered the gold standard for measuring left ventricular volume and LVEF, providing the most accurate left ventricular volume measurement [38]. In addition, cardiac MRI allows for the evaluation of myocardial properties that cannot be evaluated by echocardiography. It was reported that delayed gadolinium enhancement was observed in the left ventricular lateral wall in patients with breast cancer receiving trastuzumab [39] and that some anthracycline-treated cancer survivors experienced latent myocardial disorders with high signals in T1 mapping [40]. Cardiac MRI, a highly safe imaging modality with no radiation exposure and excellent accuracy and reproducibility, is used for diagnosing not only CTRCD but also cancer invasion and metastasis to the heart. However, it is only applicable at limited facilities due to long examination time and the medical costs involved. Therefore, it should be considered when LVEF evaluation by echocardiography is difficult.

## Echocardiographic protocol for patients undergoing cancer drug therapy

### Echocardiography before cancer drug therapy

**Fig. 1:**
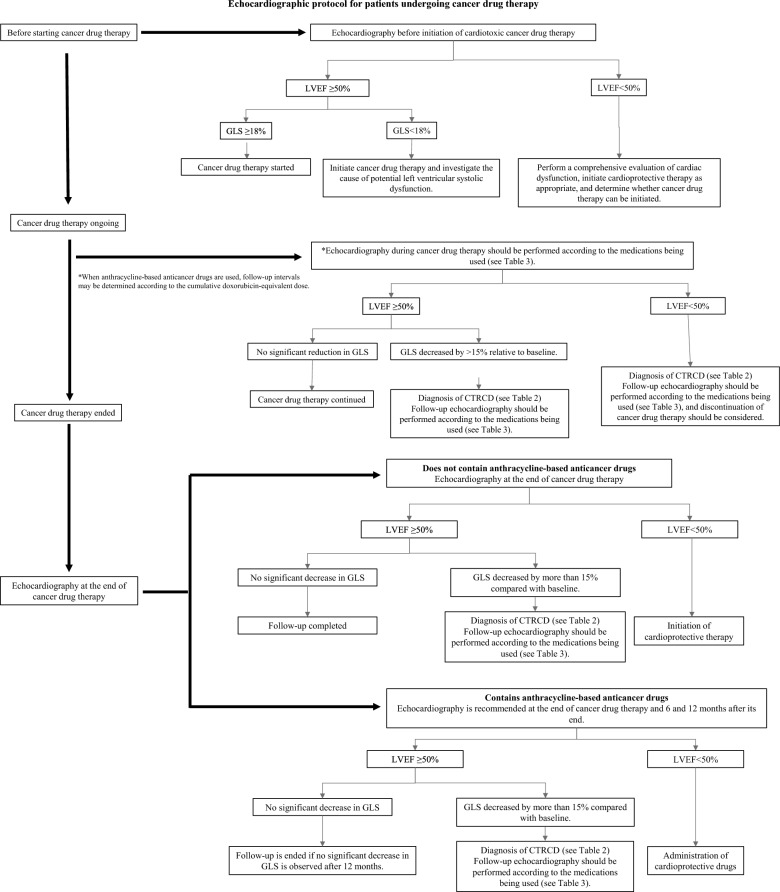
Algorithm for echocardiographic evaluation before, during, and after cardiotoxic cancer drug therapy. Baseline assessment includes measurement of left ventricular ejection fraction (LVEF) and global longitudinal strain (GLS). Cancer therapy–related cardiac dysfunction (CTRCD) is diagnosed based on changes in LVEF and/or a relative reduction in GLS of 15% from baseline. Follow-up intervals are determined according to the specific anticancer agents used and cumulative anthracycline dose. After completion of therapy, follow-up strategies differ depending on whether anthracycline-based agents were administered

The purposes of echocardiography before cancer treatment are to assess cardiovascular risk, predict potential cardiovascular complications, and obtain baseline data for early detection of cardiovascular complications during treatment; it should be performed in virtually all patients before initiating potentially cardiotoxic cancer therapy. All parameters measured in ordinary echocardiography are essential. Measuring the left ventricular ejection fraction, in particular, is of paramount importance, as it is included in the definition of myocardial disorders associated with cancer treatment. The ESC Guidelines recommend measuring LVEF using 3D echocardiography [2]. In addition, GLS is recommended as a more sensitive indicator of left ventricular myocardial disorders [2, 3]; it is also an essential parameter when available. Figure 1 shows the protocol for echocardiography in patients undergoing cancer drug therapy. Although the protocol assumes asymptomatic cases, it is recommended that echocardiography be added as appropriate if heart failure symptoms are present or cannot be ruled out or if heart failure is suspected from biomarkers (BNP/NT-proBNP, troponin) and other data. Table 3 shows durations of follow-up periods for representative anticancer drugs. Risk stratification should be performed with reference to the HFA–IC-OS risk score [41] and other information.

### Echocardiography during cancer drug therapy

#### Frequency of follow-up

During treatment with anthracycline-based anticancer drugs, echocardiograms should be performed periodically depending on the risk, as shown in Table 3. In addition, since myocardial dysfunction caused by anthracycline anticancer drugs are dose-dependent, echocardiography should be performed when the doxorubicin-equivalent dose exceeds 250 mg/m^2^ in total, even in low- and moderate-risk cases, and follow-up echocardiography should be performed as the dose increases [2, 19]. In addition, given the current situation for cardiologists to have some difficulty in accessing anticancer drug dosage information, the frequency of follow-up should be determined according to the circumstances of each facility, with a target of approximately every three months. The most important thing is to ensure that necessary follow-up is not overlooked. On the other hand, myocardial disorders caused by trastuzumab, an anti-HER2 antibody drug, often lack dose dependency compared with anthracycline-based anticancer drugs. As stated in the proper-use guidelines, it is desirable to have follow-up every three months during the treatment period [42]. The statements on such follow-ups provided in the proper-use guidelines are also applicable to new drugs. However, many of the recommendations for frequencies of echocardiography shown in the proper-use guidelines are simply repurposed from the echocardiography schedules in the study designs for various drugs, thus lacking evidence. Therefore, it is not feasible to perform follow-up echocardiography at all the frequencies shown in the proper-use guidelines. In clinical practice, echocardiographic evaluation should be considered as appropriate whenever clinically necessary, such as when clinical symptoms occur, and when chest X-rays or chest CT scans show enlarged cardiac opacity compared with pre-treatment findings. If the above criteria for CTRCD are met for the first time, the oncologist and the cardiologist will discuss the acceptability of administering cardioprotective drugs and continuing anticancer drugs. In such cases, at least in the first course, echocardiography should be repeated within 2–3 weeks. Thereafter, the cardiologist and the oncologist and cardiologist should discuss the appropriateness of initiating cardioprotective therapy and continuing anticancer treatment.

#### Examination items

The purpose of examinations performed during treatment is to detect CTRCD at an early stage and enable patients to complete cancer therapy whenever possible by administering cardioprotective drugs and adjusting the regimen. While follow-up echocardiography during anticancer drug treatment takes place at the frequencies described in the previous section, it is difficult to provide thorough follow-up to all patients because of the limitations in personnel and resources and other limitations. Therefore, the essential items are limited to assessment of left ventricular systolic function as included in the definition of CTRCD and biomarkers useful for the early detection of heart failure (BNP/NT-proBNP, troponin) [2, 43–45]. For any indicator, it is important to compare its current value with the previous and baseline values. GLS, in particular, is reportedly useful for the early detection of CTRCD; recent reports concluded that risks can be stratified based on values obtained before cancer drug therapy (Table 4).

**Table 4:** Prediction of cardiovascular events using global longitudinal strain (GLS) before treatment initiation

Authors	Journal (year of publication)	Design	Subjects (sample size)	Treatment	Cutoff value	Duration of follow-up	Prediction accuracy and results
Mousavi et al	Eur Heart J; Cardiovasc Img (2015)	Retrospective cohort	All cancer types (158 cases)	Anthracycline	No consideration (mean: 17.6%)	Median 2.2 years	GLS is an independent predictor of major adverse cardiac event (MACE) and all-cause mortality
Rhea et al	J Am Soc Echocardiogr (2015)	Retrospective cohort	All cancer types (120 cases)	Chemotherapies (various)	No consideration (mean: 15%)	Up to 3 years	GLS is an independent predictor of all-cause mortality
Ali et al	J Am Soc Echocardiogr (2016)	Retrospective cohort	Hematologic malignancies (450 cases)	Anthracycline	17.5%	Median 4.4 years	A GLS of < 17.5% is associated with a sixfold increased risk of heart failure or cardiovascular death; GLS is an independent predictor
Hatazawa et al	Circ J (2018)	Prospective cohort	Malignant lymphoma (73 cases)	Anthracycline	19%	50 months	GLS is the only multivariate independent predictor; a GLS of ≤ 19% can predict a decrease in left ventricular ejection fraction after chemotherapy
Kang et al	JACC CardioOncol (2019)	Retrospective cohort	Acute leukemia (450 cases)	Anthracycline	15.0%	Median 16 months	GLS of < 15% is the strongest predictor
Araujo-Gutierrez et al	Cardiooncology (2021)	Retrospective cohort	Various cancer types (188 cases)	Anthracycline	18.0%	≥ 3 months	The risk of developing cancer therapeutics-related cardiac dysfunction (CTRCD) increases with a GLS of < 18.0%
Thavendiranathan et al	J Am Coll Cardiol (2021)	Prospective RCT	High-risk patients (mainly breast cancer) (331 cases)	Anthracycline ± Trastuzumab	No consideration	1 year	GLS-guided initiation of cardioprotective medications prevents decreases in left ventricular ejection fraction
Liu et al	Cancer Med (2023)	Prospective cohort	Breast cancer (111 cases)	Anthracycline followed by targeted therapy	20.3% (3 months after treatment)	2 years	GLS at 3 months after treatment predicts late-onset CTRCD
Gong et al	Cardiooncology (2024)	Prospective RCT	HER2-positive breast cancer (110 cases)	Non-anthracycline	No consideration	1 year	In the low-risk, non-anthracycline-use group, prophylactic medication may be unnecessary even with decreased GLS
Rihackova et al	Sci Rep (2025)	Retrospective cohort	Malignant lymphoma (88 cases)	Anthracycline ± Radiotherapy	No consideration	Median 10 years	GLS is useful for identifying chronic cardiotoxicity even in patients with normal left ventricular ejection fraction

### Echocardiography after the end of cancer drug therapy (Table 3)

#### Frequency and duration of follow-up

In both children and adults, the use of anticancer drugs reportedly poses a risk of myocardial disorders occurring throughout life [2]. Patients receiving anthracycline-based anticancer drugs, in particular, are more likely to develop myocardial dysfunction than those receiving other agents, hence requiring periodical follow-up using echocardiography and biomarkers. It was reported that 98% of cases of myocardial disorder due to anthracycline anticancer drugs occurred within one year of treatment, the mean time to onset being 3.5 months [7]; particularly strict follow-up is required during the first six months of treatment. There is no evidence for the timing of ending the follow-up. In cases where any cardioprotective drug was administered or the anticancer drug protocol was reconsidered, due to decreased cardiac function during or after anticancer drug therapy, lifelong follow-up is recommended (target frequency: once a year, with the frequency determined according to cardiac function and clinical symptoms). In cases where no abnormalities in cardiac function were found in examination during or 6 months after treatment, the patient should be followed until 1 year after the end of treatment, if using any anthracycline-based anticancer drug, and the follow-up should be ended, provided that there were no abnormalities. For patients receiving no anthracycline-based anticancer drugs, follow-up will be ended if no abnormalities are found in the post-treatment examination. However, routine echocardiographic follow-up may not be appropriate in patients with advanced cancer of limited prognosis; therefore, their treatment should be individualized in view of the stage of cancer progression and prognosis.

#### Examination items

In follow-up examinations after treatment, as before treatment, all standard echocardiographic parameters should be assessed. The same applies to GLS measurements.

## Other complications associated with cancer therapy—echocardiographic assessment and management

### Overview

With recent advances in cancer immunotherapy, immune checkpoint inhibitors (ICIs) have become a treatment option for many types of cancer. While ICIs activate antitumor immunity, immune-related adverse events attributable to immune activation in multiple organs have attracted considerable attention, including cardiotoxicity as a potentially fatal complication. Cardiotoxicity from ICIs involves a broad range of pathological conditions, including myocarditis, pericarditis, takotsubo syndrome, non-inflammatory heart failure, and PH. The incidence of myocarditis increases with the use of multiple ICIs in combination compared with monotherapy. Although ICI-associated myocarditis is rare, occurring at an incidence of 0.5–1.2%, the relevant mortality is high at 25–50%; early diagnosis and therapeutic intervention is of paramount importance. It develops in a dose-independent manner, the time from the first ICI dose to onset is often within 3 months. Relevant symptoms include chest pain, dyspnea, palpitations, and syncope, which can develop non-specifically, hence necessitating careful monitoring [46].

### Echocardiographic findings in ICI-associated myocarditis

No echocardiographic findings are specific for ICI-associated myocarditis. Generally, acute myocarditis is characterized by wall thickening and decreased wall motion corresponding to inflammatory sites in the myocardium, narrowing of the intracardiac chambers, and pericardial effusion. Left ventricular wall motion decreases diffusely when myocardial inflammation is widespread. When the inflammation is localized, wall motion abnormalities occur locally. Even when left ventricular wall motion abnormalities are not initially apparent, deterioration may occur rapidly; therefore, serial follow-up is necessary. Evaluating right ventricular function is also important. Some cases of myocarditis predominantly affect the right ventricle; quantitative assessment of right ventricular fractional area change (FAC) and tricuspid annular plane systolic excursion (TAPSE) is recommended [47].

### Diagnostic criteria for ICI-associated myocarditis

To establish a clinical diagnosis of ICI-associated myocarditis, in addition to a significant elevation in blood troponin levels, at least one primary criterion or two secondary criteria must be met [2] (Tables 5 and 6). In the diagnosis of myocarditis, high-sensitivity troponin I (hs-TnI), which has high myocardial specificity, is increasingly recommended for measuring troponin levels; its combination with GLS changes is expected to contribute to the early diagnosis of the disease. Early reductions in GLS after ICI treatment are significantly associated with elevations in hs-TnI and are suggested to serve as an indicator of early myocardial disorders [48].

**Table 5:** Diagnostic criteria for immune checkpoint inhibitor–associated myocarditis

Pathologically established diagnosis	Multifocal inflammatory cell infiltration with evident cardiomyocyte necrosis on light microscopy
Clinical diagnosis	Excluding acute coronary syndrome and infectious myocarditis, **at least one primary criterion** or **two secondary criteria** should be met for cardiac troponin level elevations of + or lower
Primary criteria	Cardiovascular magnetic resonance (CMR) findings of acute myocarditis (in accordance with the Lake Louise Criteria as Amended)
Secondary criteria	[1] Clinical symptoms (fatigue, muscle pain, chest pain, double vision, dyspnea, orthopnea, lower extremity edema, palpitations, dizziness, syncope, muscle weakness, shock) [2] Ventricular arrhythmias or conduction disorder [3] Left ventricular systolic dysfunction (including regional wall motion abnormalities, non–takotsubo type) [4] Other immune-related adverse events (polymyositis, myopathy, myasthenia gravis) [5] Suspicious findings on CMR

**Table 6:** Severity classification of immune checkpoint inhibitor–associated myocarditis

Fulminant type	Hemodynamic instability requiring noninvasive or invasive circulatory support, high-degree atrioventricular block, or life-threatening arrhythmias
Non-fulminant type	Patients with clinical symptoms, including electrical abnormalities, but with stable hemodynamics. The condition may be diagnosed incidentally in association with immune-related adverse events. No severe signs are present despite decreased left ventricular ejection fraction
Steroid-resistant type	Cases in which myocarditis fails to remit or worsens despite steroid therapy (persistent elevation of troponin levels after exclusion of other etiologies)

### Integrated evaluation of echocardiography with other modalities

Echocardiography offers excellent convenience and is non‑invasive, but its diagnostic specificity remains limited. Although cardiac MRI is excellent for diagnosing myocarditis by T1/T2 mapping and delayed gadolinium enhancement, it may be negative in mild cases. In addition, combining FDG PET/CT examination with conventional imaging techniques may not only allow evaluation of the activity and extent of myocarditis but also be useful in assessing therapeutic response. New nuclear medicine techniques, such as ^68^ Ga-DOTATOC PET/CT, are also under development; their combination with FDG PET/CT examination is recommended for multimodal evaluations.

### Treatment of ICI-associated myocarditis

Treatment should begin with discontinuation of ICIs and initiation of high-dose intravenous corticosteroid therapy, followed by a transition to oral prednisolone. The dose should be tapered appropriately while monitoring recovery of symptoms, troponin levels, cardiac function, and arrhythmias. Although various immunosuppressive regimens have been explored as second‑line therapies for steroid‑resistant or fulminant myocarditis, the supporting evidence remains limited.

## Other complications associated with cancer treatment—echocardiographic assessment and management

### PH

PH most commonly occurs as Group 2 PH (PH due to left heart disease); [49] however, diverse etiologies are frequently encountered in cardio-oncology. Examples include pulmonary thromboembolism (PTE), PH due to cancer drug therapy, PTTM, and PH due to chronic myeloproliferative disorders (e.g., chronic myeloid leukemia) [50]. The latter two are not discussed here, as they represent forms of PH directly attributable to cancer (Group 5) [51]. Note that pulmonary angiosarcoma (classified under Category 4), lymphangioleiomyomatosis (LAM) (Category 5), and intravascular large B-cell lymphoma are also rare diseases that can cause PH. While PTE can occur as a complication due to cancer hypercoagulability in cancer-associated thrombosis (CAT), it is also known as an adverse reaction in cancer drug therapy, with cisplatin, angiogenesis inhibitors (such as bevacizumab), and ICIs being representative drugs. Regimens with these drugs can lead to venous thrombosis as a complication; modification of the treatment regimen should be considered when right ventricular overload and dysfunction due to pulmonary hypertension are severe. PH associated with cancer drug therapy is classified as Group 1 pulmonary arterial hypertension, specifically drug-induced pulmonary arterial hypertension (DPAH) [51] (Table 7). Like CTRCD, PH is sometimes encountered with multiple causes. If PH is found based on tricuspid valve regurgitation peak blood flow velocity in echocardiography, the underlying cause(s) should be thoroughly investigated, and if necessary, further extensive examination should be considered to the extent that it does not interfere with cancer treatment.

**Table 7:** Drugs associated with drug- and toxin-induced pulmonary arterial hypertension (PAH)

Drugs definitely associated with the development of PAH	Drugs possibly associated with the development of PAH
	Alkylating agents
Dasatinib	Bosutinib
Mitomycin C	Ponatinib
Carfilzomib	Bevacizumab
	Bortezomib

### Takotsubo syndrome

Patients with cancer may develop takotsubo syndrome as a complication triggered by psychological stress related to prognostic disclosure. [52] Takotsubo syndrome has also been reported in association with cancer drug therapy, reported agents include 5-fluorouracil (5-FU), capecitabine, bevacizumab, combretastatin (not yet clinically available in Japan), rituximab, tyrosine kinase inhibitors, ICIs, and osimertinib [53].

## Cardiovascular diseases following radiotherapy

### Overview

Chest radiotherapy (including the left chest and mediastinum) is performed for malignant lymphoma, breast cancer, lung cancer, esophageal cancer, and other malignancies. Radiotherapy was previously considered to have little impact on the heart and vasculature. As the number of long survivors has been increasing with advances in cancer treatment, it has become clear that cardiovascular disease can develop as a late-onset complication, attracting attention to this issue. Depending on the range of radiotherapy irradiation, pericarditis, cardiomyopathy, valvular disease, and coronary artery disease can develop, which are reported to occur in 10–30% of patients within 5–10 years after treatment. Some of them develop several decades later as a poor prognostic factor necessitating even longer follow-up [54]. Risk factors for onset of RIHD include young age, high radiation doses, and the use of anthracycline drugs (Table 8) [55], requiring special attention to these risk factors.

**Table 8:** Risk factors for the development of radiation-induced heart disease

• Irradiation to the anterior or left chest	
• High cumulative radiation dose (> 30 Gy)	
• Younger age (< 50 years)	
• High radiation dose per fraction (> 2 Gy/day)	
• Presence of a tumor involving or extending into the heart	
• Lack of radiation shielding	
• Concomitant chemotherapy (particularly anthracycline use)	
• Coronary risk factors (diabetes, smoking, obesity, hypertension, dyslipidemia)	
• History of cardiovascular disease	

### Specifics

#### Pericardial disease

Although acute pericarditis is an early complication of radiotherapy, its frequency has been decreasing due to reductions in radiation doses and the range of irradiation [56]. Chronic pericarditis can develop several weeks to several years after radiotherapy, characterized by fibrous thickening of the pericardium, adhesions, and chronic pericardial effusion, and may be followed by constrictive pericarditis. Constrictive pericarditis occurs in approximately 4–20% of cases, and its incidence increases with higher radiation doses [55].

#### Myocardial disorders

Cardiomyocytes themselves are resistant to radiation because they do not undergo cell division, but vascular endothelial injury occurs. Consequently, myocardial disease develops as a result of ischemia due to microvascular damage. As myocardial interstitial fibrosis progresses, myocardial compliance decreases, resulting primarily in impaired diastolic function, although systolic function may also be affected [57]. The cardiac conduction system can also be disordered. The incidence is reportedly approximately 10% [57], a study of myocardial disorders following radiotherapy for Hodgkin lymphoma showed that even without concomitant use of anthracycline drugs, the 25-year cumulative incidence of heart failure increased in a dose-dependent manner, from 4.4% for 0–15 Gy to 6.2% for 16–20 Gy and 13.3% for ≥ 21 Gy [58].

#### Valvular disease

Radiotherapy can cause thickening, fibrosis, shortening, and calcification of the valve leaflets and perivalvular tissue. Because this condition is more common in the left heart than in the right heart, pressure overload is considered to contribute to its development. This is distinct from rheumatic changes in that there is less degeneration of the valve leaflet tips and commissures and that calcification extends to the ascending aorta and the valve annulus. Thickening and calcification of the aortic-mitral valve fibrous continuum are characteristic of a history of radiotherapy, and their degrees are related to the prognosis [59]. Because the valve shortens, regurgitation is more common than in stenosis. Clinically significant valvular heart diseases reportedly occur at incidences of approximately 1%, 5%, and 6% at 10, 15, and 20 years, respectively, after radiotherapy. Specifically, the incidence rates at 20 years after radiotherapy were approximately 45% for mild aortic regurgitation, 15% for moderate to severe aortic regurgitation, 16% for aortic stenosis, 48% for mild mitral regurgitation, and 12% for mild pulmonary regurgitation [55]. When considering surgery for severe valvular heart disease, there are high risks associated with open-heart surgery, such as mediastinal adhesions, pulmonary fibrosis, and ascending aortic calcification; in cases of aortic stenosis, transcatheter aortic valve replacement may also be considered [54, 60].

#### Coronary artery disease

Coronary artery disease due to radiotherapy results from arteriosclerosis promoted by endothelial disorder. The condition typically becomes apparent 15–20 years after treatment and is more likely to occur in the young than in the elderly [61]. A study on coronary artery disease after radiotherapy for Hodgkin lymphoma reported that 10% of patients had coronary artery lesions 20 years after treatment [62]. An increased number of coronary risk factors increases the likelihood of disease progression; for example, in left-sided breast cancer, which is treated by radiation to the left breast, lesions are often found in the left main coronary artery or its proximal region [55]. Open-heart surgery carries high risks, and catheterization treatment is reportedly associated with a higher incidence of restenosis than typical arteriosclerotic lesions, requiring careful judgment for treatment indications.

### Follow-up after radiotherapy

**Fig. 2:**
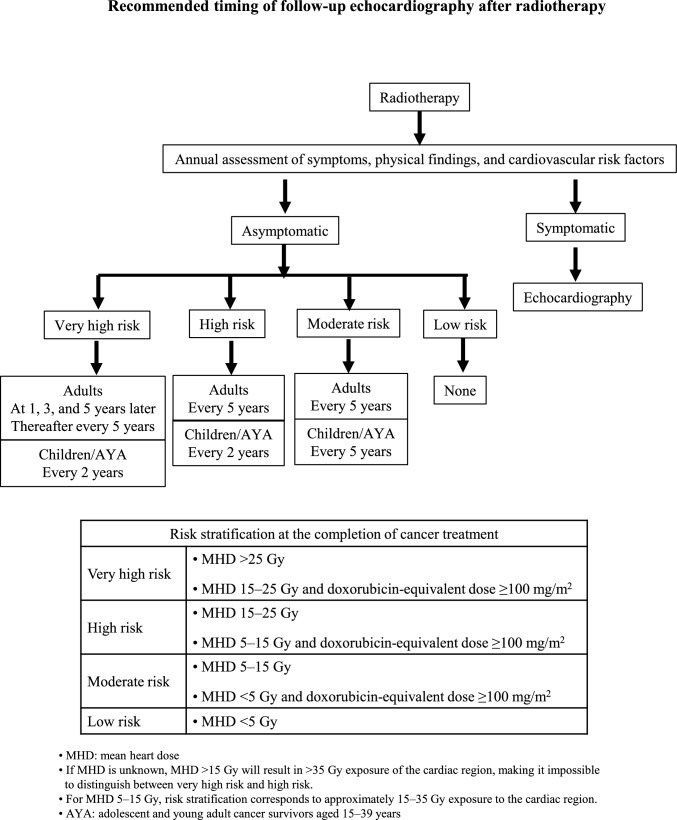
Risk classification at the completion of radiotherapy is based on mean heart dose (MHD) and cumulative doxorubicin-equivalent dose. Patients are categorized into very high-, high-, moderate-, or low-risk groups. Follow-up strategies differ between adults and children/adolescents and young adults (AYA). Periodic echocardiographic surveillance is recommended according to the assigned risk category and the presence or absence of symptoms

Routine echocardiography is not always required before or during radiotherapy. After treatment, as described above, however, radiation-induced cardiac disorders are manifested often several years, or even more than 10 years later; emphasis should be placed on periodic health checkups and risk factor assessment (Fig. 2). Although radiation to the heart has recently been decreasing, periodic long-term follow-up with echocardiography is necessary depending on the risk level [2, 63].

## Practical considerations for echocardiographic measurements in clinical practice

### Advances in cardio-oncology and roles of echocardiography

To date, echocardiographic LVEF and GLS have been established as essential indicators for monitoring CTRCD. Changes in these indicators are important factors influencing treatment strategies for patients with cancer. To ensure that patients with cancer receive safe and appropriate treatment, those performing echocardiography must strive to ensure measurement accuracy. Published in 2019, the “Japanese Society of Echocardiography Practice Guidance for Echocardiography in Cardiac Disorders Associated with Cancer Drug Treatment” [1] describes the points to note and accuracy standards for echocardiography practice in clinical settings. However, since then, new cancer drug therapies, particularly ICIs, have become widely used in clinical practice, leading to a deeper understanding of the pathology of associated cardiotoxicity and methods of its evaluation. In addition, technological innovations, including artificial intelligence (AI), are dramatically improving the accuracy and efficiency of echocardiographic measurements. Furthermore, new recommendations have been presented by international academic societies, including the ESC Guidelines published in 2022 [2]. The following section reviews the recommendations of the 2019 guidance and describes methods for further increasing the accuracy, reproducibility, and utility of echocardiography in clinical practice, with mention of recent findings.

### Importance of monitoring indicators and accuracy control for CTRCD

While LVEF and GLS as measured by echocardiography are already established as essential indicators for monitoring CTRCD, caution is needed regarding their reproducibility (measurement variability). Recent studies have reported that the intra-observer and inter-observer variability for LVEF is approximately 5–10%, [64, 65] and that the variability of GLS measurements is approximately 5% [66]. Since GLS is a more useful indicator than LVEF for the early detection of CTRCD and has superior reproducibility, its measurement is recommended in the ESC and other international guidelines for the purpose of early detection of myocardial disorders. It should be noted, however, that many reports on their reproducibility came from facilities with sufficient experience and skills in echocardiography; however, the same level of reproducibility may not be achievable in general clinical settings, such as community hospitals or clinics. Special attention should be paid to the fact that a 10% error in LVEF can affect the diagnostic criteria for CTRCD. In clinical practice, it is necessary to make a comprehensive determination taking GLS changes into account and keeping in mind that small LVEF changes (e.g., < 10%) may fall in the range of measurement errors.

### Maintenance and education in the ultrasound examination room for echocardiography accuracy control (Table 9)

**Table 9:** Recommendations for equipment management and quality control in the echocardiography laboratory

It is recommended that echocardiography laboratories and equipment be maintained and inspected in accordance with established guidelines	
In patients with a history of echocardiography, previous and baseline measurements and images should be reviewed prior to the examination	
Echocardiographic measurements should be verified for consistency with visual assessments by an experienced examiner	
Periodic assessment of intra- and inter-observer variability in echocardiographic measurements is recommended to ensure measurement accuracy	
Echocardiographic still images and cine loops should be stored on an image server to allow timely access and re-measurement	

First, to ensure that the ultrasound diagnostic equipment functions properly for measurements to be taken, the echocardiography laboratory and equipment must be maintained and inspected in accordance with the guidelines [1]. To ensure the accuracy of echocardiographic measurements, it is recommended that the facility strive for accuracy control by checking intra- and inter-examiner errors in LVEF and GLS measurements at least once a year. In addition, staff education is essential for ensuring the accuracy of echocardiographic measurements. Less experienced examiners should be trained by highly experienced individuals, such as society-certified echocardiography specialists and technicians, to ensure that the examiners can perform measurements with the same level of accuracy as the certified staff. It is desirable that still images and videos from echocardiography be stored in an image server in preparation for referencing upon any demand and that equipment be available to allow timely re-measurements.

### Practical considerations in echocardiographic measurements (Table 9)

Minimizing measurement errors for echocardiographic indices is indispensable for determining the appropriate treatment strategy. To provide accurate measurements, the following points must be considered when conducting the examination:

#### Checking previous measurement values and baseline measurement values

In patients with a history of echocardiography, their previous and baseline measurements and images should be checked before the examination. To this end, be sure to record baseline videos. If a person with sufficient experience in echocardiography determines that the previous measurement is inappropriate compared with the image, they will perform a re-measurement and contact the attending physician.

#### Measurement by the same examiner

If possible, the same examiner should perform serial examinations in the same patient. This does not apply, however, if it is difficult due to staffing limitations at the facility.

#### Measurement using the same equipment

GLS measurements, in particular, may vary among different vendors [67]. Standardization efforts by the taskforces led by ASE and EACVI have improved the variability in analysis results among different vendors [68]; however, for measurements in the same patient, it is desirable to use ultrasound diagnostic equipment of the same manufacturer whenever possible. This does not apply, however, if it is difficult due to resource limitations at the facility.

#### Verification of the validity of measured values

Echocardiographic measurements should be checked for consistency with visual assessments by a highly experienced examiner. Whenever possible, measurement validity should be confirmed by an echocardiography specialist or certified sonographer in addition to the examiner. In cases with history of echocardiography, check whether the changes in echocardiographic measurements are consistent with the visual changes on the images.

### Making the best use of AI

Recent studies have reported that AI-based automated measurements demonstrate higher accuracy and better reproducibility than those of manual measurement in measuring LVEF and GLS [69–72]. Introducing AI-based automatic measurement can shorten measurement times and reduce the workload on laboratory technologists thereby streamlining workflows and enabling more examinations to be performed efficiently. On the other hand, the validity of the measurement results should be managed by echocardiography specialists and certified technicians who are able to make comprehensive judgments while checking the findings against imaging data and patient information. It is also recommended to use AI measurement software with fully verified measurement accuracy.

### Making the best use of 3D echocardiography

3D echocardiography allows more accurate calculation of LVEF than 2D echocardiography [73]. Although 3D echocardiography has not yet been fully established in routine clinical practice in Japan due to the costs of introducing measurement software and equipment, its proactive use helps improve the accuracy of LVEF measurement.

### Echocardiography laboratory manager

Up to this point, we have described the educational requirements, system considerations, and equipment controls essential for accuracy control in echocardiography. Most importantly, the echocardiography laboratory should be managed by a cardiac echocardiography specialist or certified sonographer who possesses the requisite knowledge and expertise. With the emergence of new anticancer drugs and the compilation of relevant knowledge, echocardiographic evaluation in CTRCD continues to advance. The manager must ensure the responsible operation of the echocardiography laboratory while continuously updating their professional knowledge.

## Data Availability

This article does not contain any new data generated or analyzed by the authors. All information presented is based on previously published literature and existing clinical guidelines.
